# Selections

**Published:** 1892-10

**Authors:** 


### *SELECTIONS.*

---

### Treatment of Infantile Paralysis.

The treatment of the acute stage of the disease consists in the administration of antipyretics: Antipyrin, bromide of sodium, salicylate of sodium and ergot, if the disease can be recognized in this stage. When the acute stage is over, then the tonics are indicated, iron, quinine and strychnia in the beginning, cautiously. In some cases cod-liver oil and the syrup of hypophosphites are extremely useful. The most important agent in the treatment of this affection is electricity, and the constant galvanic current should be used as soon as the fever has subsided, and should be made to pass through the seat of the lesion in the cord. The electrodes used should be large, and the current should be mild and its duration should be five to ten minutes. At a later period of the disease, when atrophy of the muscles has set in, the interrupted galvanic current should be used to the paralyzed muscles, of the least strength that will produce muscular response, and each paralyzed muscle or each group of muscles should be made to respond to this electrical stimulus not more than once or twice at each séance. As the muscles improve in tonus, less strength of current will be necessary, and after a time the muscles respond to the Faradic stimulation.

The electricity should be applied by the physician in person, much of the want of success in the treatment of this disease being due to the non-observance of this rule. It is a common experience for me to be consulted by the friends of these patients and when I advise the use of electricity to be told that they have been using it, and it has been of no value; and upon further investigation find that they have themselves been applying the current from a Faradic battery that was incapable of producing the slightest muscular reaction. It is the rule that the Faradic currect will not produce muscular response, hence its use under such circumstances even by the physician is useless.

Along with the electricity there should be used massage of the paralyzed limbs, and if the nutrition of the muscles does not improve under the stimulus of electricity and massage, then stimulating applications may be made to the surface of the limb, and for this purpose we prefer to use the capsicum plaster; placing around the paralyzed limbs strips of plaster about from ½ to ¾ of an inch in diameter, which are to be worn for two or three days and then renewed, and if after a reasonable space of time, the limbs do not improve under this treatment, then I advise the use of deep injections of strychnine, preferring the nitrate of strychnine as less irritating, using full dose, and giving one injection every day.

This treatment must be persisted in for months, even though but little if any improvement is at first noticed, but even the most unpromising cases will occasionally yield to the treatment if perseveringly pursued. The occurrence of deformities will require appropriate surgical treatment, but should not cause us to stop the use of remedial measures. During the progress of this treatment, the child should be encouraged in every possible way to use the paralyzed muscles, and, of course, every possible attention should be given to the hygiene of the patient, for that condition of living that results in the best general vigor will be of the most service in correcting this special disturbance of nutrition.—DR. D. R. BROWN, in *Med. and Surg. Reporter*.

---

### THE PROPER COURSE TO PURSUE WHEN A WOMAN WITH SYPHILIS CONSULTS YOU.

Professor Fournier has just given in one of his remarkable lessons the line of action to follow when consulted by a syphilitic woman. This varies with the social status of the patient. If *irrégulière*, that is to say a woman who is kept or a prostitute, she must be told at once the nature of her ailment so as to put her on her guard against the danger of infecting others if she persists in maintaining relations with them. If on the contrary it is a married woman the line of conduct is much more delicate. To declare rudely that she is affected with syphilis is to risk, if the husband is the culpable one, causing domestic trouble, and separation in the family. If one simply gives a prescription without saying anything, this usually amounts to nothing, for she does not fail to instruct herself upon the real nature of the drugs employed, and thus learns the truth. Theoretically it is better to place the burden on the husband of keeping from the wife so far as is possible the nature of the disease which she has received from him. The husband must thus be taken into the case and entrusted with the direction of the care to be taken and the carrying out of the treatment. For this reason Professor Fournier does not advise giving the patient at the first visit a prescription which could awaken suspicion. A local treatment without mercury should suffice. The patient must be told that there are certain points in the case which do not appear quite clear, and for which reason a conversation with the husband will be necessary, or that there are certain things you wish to recommend to the husband, and ask to have him sent to see you. If the woman is innocent, that is to say if she has had relations with her husband alone, she will readily comply with your request. From this time on you have only to come to an understanding with the husband, keeping as much as possible from the knowledge of the wife the real nature of her complaint. However too much secrecy cannot be expected, for after a certain period of this prolonged and mysterious medication the woman begins to suspect the truth, but the object has already been attained, scenes of violence have been avoided, which in the commencement would have been precipitated by a sudden discovery of the true nature of the disease, and usually regrettable separations and divorces are avoided. But to act as we have suggested we must be quite sure of our premises. We must be assured that it is from the husband alone and not from some other that the contagion has come, for in the opposite case it would be the same as saying to the husband, "Your wife has syphilis, and as you haven't it, she must have contracted it from some one else." So as not to commit such a blunder care must be taken in speaking to the husband before having the full assent of the wife. If she is innocent she will have no reason to oppose the conference, if she is guilty, that is to say if she has had relations with other persons she will without doubt take you at once into her confidence.—*Med. and Surg. Reporter.*

### TWO HUNDRED CASES OF ECLAMPSIA.

At a recent meeting of the Berlin Medical Society, Olshausen (*Am. J. Med. Scien.*) gave the results of his study of two hundred cases of eclampsia.

As is often observed in hospital practice, his cases came in series, the larger portion of them between September and February. Most of them were primiparæ, and especially those older than the average. Twin pregnancy was also a frequent cause. The frequency of eclampsia Olshausen estimates as greater than twenty-five per cent. of all cases. Thirty per cent. of premature labors, he thinks, are caused by eclampsia. In five cases out of two hundred, pregnancy was not interrupted by this complication. It is rare for eclampsia to persist after a brief period of the acute disorder.

As prodromal symptoms, were observed headache, and more important, gastric pain with frequent vomiting. Amaurosis was rarely observed. In three cases an aura was present.

Albumin was almost invariably present in the urine. Casts were often found, œdema rarely, and icterus was observed twice.

Twenty-five per cent. of the 200 perished, and *post-mortem* examinations were obtained in thirty-seven cases. Acute affections of the kidneys were found in twenty-two of these; chronic interstitial nephritis in four. The remainder showed a mixture of acute and chronic diseases; in two cases the kidneys were unchanged. In six cases a ureter was dilated, but not to a pathological extent. Nine patients died before the end of labor, and labor was terminated by Cæsarean section in one patient, and the life of the child was preserved. Most fatal cases terminated in four days after the first convulsions, although convulsions occurred as late as thirtieth or fortieth day after labor. The complications causing death were sepsis and pneumonia, eclamptic patients being especially susceptible to septic poison.

Regarding prognosis, it is often favorable where convulsions begin soon after the birth of the child. Frequent convulsions, rise in temperature, small and frequent pulse are signs of a fatal issue. Twenty-eight per cent. of the children born at term died

This result is sometimes owing to morphia-intoxication where large doses of the drug have been given.

As regards the treatment of eclampsia, Olshausen depends upon morphia, chloral, and the use of forceps. Version is contra-indicated, and chloroform should be used only where the convulsions recur regularly. The intoxication theory of eclampsia seems most probable, and an interesting analogy is shown between the lesions of eclampsia and those of sublimate-intoxication.—*Archives of Gynecology.*

---

### ON THE RELATIVE VALUE OF PERINEAL AND SUPRAPUBIC LITHOTOMY.

Dr. Wilhelm T. Lindenbaum, Jaroslavl, Russia (*Meditzinskoiè Obozrénïè*, No. 2, 1891, p. 133), in the course of the last nine years, has made seventy-nine perineal lithotomies in children under fifteen years of age, with two deaths, and thirty-two in adults, with eight deaths. Besides during 1890 he performed ten supra-pubic lithotomies in patients aged from eight to fifty-two years with one death (the fatal case referred to a man of fifty-two with pulmonary tuberculosis and fatty heart). The high operation was conducted after the following rule:

1. All instruments were sterilized.
2. Colpeurynter was introduced into the rectum.
3. The bladder was filled up with 250 ccm. of a salicylic solution.
4. Drainage was inserted (no vesical suture being employed).
5. The patient was kept on his abdomen for from eight to ten days.
6. The dressing was changed once daily.

The urine began to flow through the urethra on an average on the twentieth day, the wound soundly healing on the thirtieth. As far as young children are concerned, suturing the bladder is thought to be very difficult, and, on the other hand, quite superfluous, since a healthy urine does not irritate the wound. The author's general corollaries are as follows:

1. Perineal lithotomy in early childhood represents a safe operation and gives excellent results. A relatively enormous percentage of death in old age can be explained by the coexistence of grave complications about viscera, especially kidneys.

2. Suprapubic lithotomy does not offer any important advantages over the perineal operation. The mortality remains yet very high, even in children.

3. Still, speaking generally, in the presence of stones measuring above 2 cm. in diameter, the high section should be preferred, but in cases of smaller calculi perineal lithotomy should be performed.—*Annals of Surgery.*

---

### DIGITALIS IN CASES OF AORTIC REGURGITATION.

The action of digitalis, therefore, in slowing the heart is not hurtful in aortic regurgitation, but beneficial. But apart from its action in slowing the heart, which is a mere accidental or secondary effect, digitalis possesses pre-eminently the properties belonging to what is called the digitalis group of remedies. It increases the elasticity of the muscular tissue, so that this extends and contracts more completely and perfectly, and as all the blood passes more frequently through the heart than through any other muscle, this action is specially exerted upon it, and is manifested at a time when the other muscles are practically uninfluenced. The importance of such an action on a failing heart cannot be overestimated; in all cases of ruptured compensation in mitral incompetence we are accustomed to rely upon it with the utmost confidence, and are rarely disappointed. Why should we have misgivings in aortic incompetence? In the early stages of aortic incompetence the heart is well fed, every part of it is flushed with blood, which, from the increased size of the blood-wave and the position of the coronaries, must beat first, at all events, at an abnormally high pressure; the nutrition of the heart is specially well provided for, there are no symptoms, and no treatment is required. When, from any cause, however, the compensation is ruptured, an aortic heart will be found as amenable to the beneficial influence of digitalis as any other failing heart, but larger doses are required, but little influence is produced by less than three times as much as would suffice for a mitral heart. Even should the pulse under treatment become abnormally slow, which is not at all usual, and certainly not needful to secure benefit, we may rest assured that excessive regurgitation is not then promoted; and though sudden death is not at all unlikely to happen in a badly-compensated aortic heart, whether it is treated with digitalis or not, digitalis is never to blame for this. On the contrary, the judicious use of digitalis is the most efficacious treatment in all cases of failing heart, whether that failure be accompanied by aortic or mitral regurgitation. In failure dependent on arterio-sclerosis alone, the tonic influence of digitalis on the heart is hindered unless we combine it with some drug which unlocks the arterioles, and so prevents an increase of the blood pressure, already abnormally high.—*Therap. Gazette.*

---

### One Thousand Cases of Labor without a Death.

This is the result obtained at the Preston Retreat, by Dr. Joseph Price:

In this series of one thousand cases, a number of complicated labors were dealt with successfully both as regards the mother and child. Many feeble and impoverished subjects were treated. The patients are admitted two weeks before labor, and remain four weeks after labor. The toilet begins on admission with a bath, a laxative, and clean clothing. Until the occurrence of labor, the woman takes two soap baths a week, the bowels are kept soluble, and the kidneys are watched. When the premonitory symptoms of labor appear, a thorough bath is given, superintended by a careful nurse, an enema is administered, and the vagina douched with 1-2000 solution of bichloride of mercury, and the woman is given clean clothing, and placed in a clean delivery room. The nurse makes a toilet and the physician makes a toilet before and after entering the room. As a rule only one examination is made. For some six years there has been no meddlesome midwifery. It is only in complicated labors that repeated examinations are made. But two vaginal douches are given, one before and the other after labor. It has never been necessary to repeat the douches. After the final douching, a pint or so of the fluid is poured over the thighs and external genitals, and the pad is applied. This is changed four to six times a day for the first four days. There is never a perceptible odor. The mothers nurse their children without exception, and the mortality among the infants is very low. After labor the bowels are kept soluble. The patients are well fed before and after labor. They remain in bed usually eleven days and then go to the convalescent ward.—*Columbus Med. Jour.*

---

### THE BACILLUS OF ECLAMPSIA.

A very interesting, and probably practical, discovery is that of eclampsia bacillus by Dr. Gerdes, of Halle. Sometime ago Gerdes called attention to the existence of a bacillus which he thought played an important part in the etiology of this disease. He now states that he has demonstrated the existence of colonies of this bacillus in the body of a patient who died from eclampsia. The bacilli were found in the lungs, in the septum ventriculorum, in the placental areas, in enormous quantities, and in the liver and kidneys in smaller numbers. They did not exist in the spleen.

Gerdes was able to obtain pure cultures of the bacillus. This proves, he held, that eclampsia is an infectious disease. Numerous interesting questions have yet to be discussed in this direction, but Gerdes believes that the bacilli enter through the cells of the decidua as a result of endometritis, and are forced by the uterine contraction into the sinuses, and thence into general circulation. The following were the conclusions of his paper: 1. The eclampsia bacillus is the only cause of eclampsia and is found in no other disease. Infection occurs through the uterus, most probably in consequence of an existing endometritis. 2. The causes of other forms of convulsions during labor are decidedly different, post mortem, from those giving rise to true eclampsia. 3. Eclampsia is an anatomically limited and characteristic disease. 4. The existence of the well-marked lesions found in eclampsia is not sufficiently explained by the mere presence of the specific bacillus. Most likely the direct or indirect causes are the toxin of the eclampsia bacillus.—*Berlin Correspondent of the Medical Record.*

---

### GONORRHŒA.

My idea about gonorrhœa is that we should treat it just as we do an acute abscess; that is by drainage, with just as much asepsis as possible. Let the urethra hang downwards, with a bag of some sort loosely attached to it to catch the discharge. Do not have it tightly bandaged or plugged up with cotton. In a specific urethritis, where the inflammation has extended into the deeper layers of the epithelium, the various bichloride and zinc injections are not likely to do much good. In an ordinary, non-specific urethritis, however, where the inflammation is superficial, you can inject with good results. There are certain remedies which will increase the amount of urine and render it aseptic, so that you can use the bladder as an irrigator in gonorrhœal inflammation. Among these remedies is boracic acid, or, better still the oil of gaultheria. This drug will absolutely sterilize the urine. You can give five or six drops every three or four hours. During the latter stages of the inflammation, mild solutions of the sub-acetate of lead can be used, which will help keep the urethra clean. A syringe with a short nozzle should be employed, so as to only just enter the meatus. The injection should be of moderate size, so as not to overdistend the urethra. During the acute stages, I instruct my patient to take a warm sitz bath night and morning. When the acute stage has subsided, the administration of some of the so-called blennorhetics will be found beneficial.

I have found the capsules containing Santal-midy, a preparation made from sandal-wood, a very good thing. I consider these superior to the old-time cubeb preparations, which are liable to disturb digestion.—*Dr. Jno. A. Wyeth, New York.*

### THE TREATMENT OF BURNS.

Von Bardeleben (*Deutsche Medicinische Wochenschrift*) states that no specific for the relief of pain of burns has as yet beet. discovered. The author's present method of treatment is as follows:

After carefully cleaning the burned area, it is irrigated either with a three per cent. carbolic solution or a thirty per cent. salicylic acid solution. Sublimate lotions are avoided because of the great pain they produce.

After all the blebs are opened the entire surface is covered with powdered bismuth; over this cotton is applied. This absorbs any discharge, and fully protects the burned surface from the air. The cotton may be sprinkled with a powder composed of equal parts of bismuth and starch.

This dressing may be allowed to remain from one to three weeks, according to the case. In cases of burns about the face it is only necessary to cover the burned parts with the powder, the bandage being omitted because of the discomfort it occasions.

Under this treatment the author has seen children recover where two-thirds of the body were involved.

Von Bardeleben thinks the bismuth probably exerts some influence in preventing intestinal complications, as in one hundred cases treated in this manner only two had blood in their stools. In using the bismuth there is no danger of intoxication from absorption, even in cases where it is extensively applied. By the antiseptic treatment secretion is greatly diminished.—*Therap Gazette.*

---

### "HEART FAILURE" NOT A CAUSE.

The clerk of the Board of Health of Syracuse, says *Insurance*, recently refused to issue a burial permit on the certificate of the physician attending the deceased, which simply gave "heart failure" as the cause of death. The physician, when informed by the undertaker of the clerk's refusal, declined to give any more definite account of the case for the benefit of the board than was contained in the words heart disease. This resulted in the board, at its next meeting, adopting a resolution to the effect that physicians in the future will have to specify the direct cause of death, and that "heart failure," "asthenia," and such generalities will not answer the purpose. This is sensible. To say that a person died from "heart failure" is to say no more than that the person died because his or her heart stopped beating, and to assign "asthenia" as the cause of death is simply to affirm that the person died by reason of inability to live. The matter is an important one not alone for the sake of having mortality statistics so classified as to give valuable information to students of social problems, but in order that crime may not be covered up by smooth ambiguities. Either of the terms above mentioned would describe a death by criminal abortion, or by poisoning, or by typhoid fever, or by drowning, or by measles, or death by any other cause. It amounts to nothing more than saying that the person died.—*Exchange.*

---

### GUNSHOT WOUNDS OF THE BRAIN.

Following is the summary of an interesting paper on this subject, read before the last meeting of the American Medical Association, by Dr. C. E. Ruth, Keokuk, Iowa:

1. That a ball can be followed in its course through the brain.

2. Having been followed to its point of impaction on the opposite side of the skull, a trephine disc should be removed, drainage be established and the ball removed if possible.

3. That the probe is best which gives the greatest resistance to penetration with least possible lateral friction on its shaft by he collapsed canal.

4. That hemispherical front, porcelain tip and aluminum shaft answers the indications for lead detection (resistance if of proper size, weight, etc.,) required in a probe better than anything else.

5. That one intending to follow balls through the brain should thoroughly familiarize himself with the resistance the normal brain offers to penetration by the probes he expects to use, that he may know when he is applying force within safe limits.

6. That he should frequently grasp and remove balls and pieces of bone with the forceps of his choice on the cadaver, before attempting it on the living human subject.—*Jour. Am. Med. Association.*

---

### THE TREATMENT OF LEG-ULCERS.

At a meeting of the Philadelphia County Medical Society T S. K. Morton recommended the following treatment of leg-ulcers: The surroundings are thoroughly cleansed with soap, brush and water, and, if necessary, shaved. Then a douche of simple water is applied, followed by one of sublimate solution (1: 1000) if the ulcer is foul, inflamed, or otherwise contaminated. Next, a spray of hydrogen dioxide (15-volume solution) is employed. When active effervescence has ceased, the surface is gently irrigated with simple water. The ulcerated area is then covered with strips of protective. A dressing of gauze or butter-cloth wrung out of sublimate solution is next applied; over this the bandage, without reverses. The treatment should be repeated daily, or on alternate days, until the wound presents a healthy appearance, and then with diminishing frequency.—*Med. News.*

---

### TO CONTROL THE SEX.

We had always thought that the cause of sex would be one of the last things which science would discover. However, all difficulties now seem to be removed. In a late *New York Medical Journal* a Dr. Keefe thus explains:

"When a given couple are having an excess of girls, and it is desired to have boys, they must refrain from all sexual intercourse, the female must wean her child, and live almost continuously out of doors, having gentle exercise, but not carried to the point of fatigue. She must be relieved of all, or nearly all, of her household cares, have a good rich diet of milk, rare-done meat, wine, fruit in moderation, amusements, and everything calculated to promote cheerful and happy thoughts. Her husband must have a more sparse diet and hard work. When male children are in excess, this treatment, except the paragraph referring to lactation, may be applied to the husband. Referring to animals, I would apply the rule in the same manner, and in addition would allow the male, if the stronger, to have connection with other animals, while the female out of which it is desired to breed a male is resting."

---

### SURGICAL TREATMENT OF FOREIGN BODIES IN THE BRAIN.

Dr. H. B. Luhn (*University Medical Magazine*) says:

For the treatment of a foreign body in the brain, from the statistics on the subject, the following is advised: (1) Gentle probing, to detect the presence of the foreign body; no force is to be used. (2) Removal of the fragments about the wound of entrance, and thorough disinfection of the latter. (3) Avoidance of prolonged and elaborate search, should the bullet not readily be found. (4) Drainage and the application of a most careful antiseptic dressing. If there is any bleeding, this can be controlled by an antiseptic iodoform gauze tampon, which will at the same time serve very well for drainage. (5) After a careful antiseptic dressing, apply cold to the head. Open the jugular and bleed, in case symptoms of encephalitis develop and are not controlled by careful irrigation and redressing. (6) Place the patient at absolute rest; give a light and nutritious diet, stimulants if necessary.—*American Lancet.*

---

### SALOL IN DIARRHŒA.

In the *Therapeutic Gazette*, Aug. 15th, Dr. M. H. Fussell gives his experience in the treatment of diarrhœa (especially that of children) with salol. His results correspond perfectly with our own. He concludes:

1. Diarrhœa due to dietetic errors, and that which is common in adults and infants in summer, is well controlled by the administration of salol and bismuth or chalk.

2. Opium is rarely necessary where salol is used.

3. Salol controls the abdominal pain equally as well as opium.

4. It is perfectly safe, having no bad after-effects.

5. It is especially useful in the treatment of the diarrhœa of children.

6. It is of no value in dysentery.

7. It constantly corrects the fœtor of the stools.

---

### ON THE TREATMENT OF HEPATIC COLIC BY ANTIPYRIN.

The *Journal de Médecine de Paris* quotes from the May number of *Les Nouveaux Remèdes* an article of Strisover on the successful employment of repeated doses of antipyrin in cases of hepatic colic. Between the paroxysms he prescribed salol and salicylate of sodium three time a day in the dose of eight grains. This treatment was continued as long as possible. The salicylate of sodium was dissolved in Vichy water. By means of the salicylates, Strisover believed himself to be able to inhibit the formation of other calculi, and by the administration of 7 grains of antipyrin, as needed, given during the paroxysm, he was able to relieve the pain.—*Therap. Gazette.*

---

### TREATMENT OF BOILS.

Dr. Craig, in the *Reporter*, recommends the following treatment for boils :

| | | |
|---|---|---|
| ℞. | Tinct. ferri chlor., . . . . | gtt. xv |
| | Liquor potassii arsenitis, . . | gtt. iii |
| | Aquæ, . . . . . . | f ʒ ss |

Take at a dose after each meal.

Use locally, carbolized camphor, and a poultice when there is much inflammation. Also give small doses of Epsom salts to prevent constipation.—*Brooklyn Med. Journal.*

---

### SPECIAL TONIC (BELLEVUE HOSPITAL).

| | | |
|---|---|---|
| ℞. | Sulph. quinine, . . . . | grs. 30. |
| | Tinct. nux vomica, . . . | *m* 160. |
| | Tinct. chlor. iron, . . . | *m* 160. |
| | Dilute phosphoric acid, . . | fl. ℥j. |
| | Syrup to make, . . . | fl. ℥iv. |

M. Dose, a teaspoonful.

—*Lancet-Clinic.*

